# Deep Learning for Automated Measurement of Total Cardiac Volume for Heart Transplantation Size Matching

**DOI:** 10.21203/rs.3.rs-3788726/v1

**Published:** 2023-12-28

**Authors:** Nicholas A. Szugye, Neeraja Mahalingam, Elanchezhian Somasundaram, Chet Villa, Jim Segala, Michael Segala, Farhan Zafar, David L.S. Morales, Ryan A. Moore

**Affiliations:** Cleveland Clinic Foundation; Cincinnati Children’s Hospital Medical Center; Cincinnati Children’s Hospital Medical Center; Cincinnati Children’s Hospital Medical Center; SFL Scientific; SFL Scientific; Cincinnati Children’s Hospital Medical Center; Cincinnati Children’s Hospital Medical Center; Cincinnati Children’s Hospital Medical Center

**Keywords:** Artificial Intelligence, Deep learning, Heart Transplant, Congenital Heart Disease, Size Matching, Imaging

## Abstract

**Background:**

Total Cardiac Volume (TCV) based size matching using Computed Tomography (CT) is a novel technique to compare donor and recipient heart size in pediatric heart transplant that may increase overall utilization of available grafts. TCV requires manual segmentation, which limits its widespread use due to time and specialized software and training needed for segmentation.

**Objective:**

This study aims to determine the accuracy of a Deep Learning (DL) approach using 3-dimensional Convolutional Neural Networks (3D-CNN) to calculate TCV, with the clinical aim of enabling fast and accurate TCV use at all transplant centers.

**Materials and Methods:**

Ground truth TCV was segmented on CT scans of subjects aged 0–30 years, identified retrospectively. Ground truth segmentation masks were used to train and test a custom 3D-CNN model consisting of a Dense-Net architecture in combination with residual blocks of ResNet architecture.

**Results:**

The model was trained on a cohort of 270 subjects and a validation cohort of 44 subjects (36 normal, 8 heart disease retained for model testing). The average Dice similarity coefficient of the validation cohort was 0.94 ± 0.03 (range 0.84–0.97). The mean absolute percent error of TCV estimation was 5.5%. There is no significant association between model accuracy and subject age, weight, or height. DL-TCV was on average more accurate for normal hearts than those listed for transplant (mean absolute percent error 4.5 ± 3.9 vs. 10.5 ± 8.5, p = 0.08).

**Conclusion:**

A deep learning based 3D-CNN model can provide accurate automatic measurement of TCV from CT images.

## Introduction

Total Cardiac Volume (TCV) based size matching has emerged as a novel technique to compare donor and recipient heart size in pediatric heart transplant. Traditionally, heart transplant size matching has been performed by comparison of donor and recipient weight, though the inaccuracy of this method has been revealed in recent years[1–4]. TCV-size matching is performed by directly comparing the whole heart volume on Computed Tomography (CT) scan or echocardiogram of donor and recipient. By precise measurement of the potential space within the recipient chest cavity, TCV may increase the donor pool for an individual recipient by broadening the acceptable donor size, enabling more heart transplants [5–7].

A major drawback of direct TCV comparison for size matching is reliance on segmentation by a trained imaging specialist, limiting its widespread adoption. Image segmentation takes on average 30 minutes to complete and requires specialized software and training unavailable at most transplant centers. Artificial Intelligence (AI) technologies are now available that enable accurate measurement of body structures within 3D image datasets by training a custom 3D-Convolutional Neural Network (3D-CNN) to recognize specific voxels of a 3D dataset[8]. Use of 3D-CNN model to calculate TCV from a CT scan is an attractive means to measure TCV more quickly, limit interrater variability, and enable directly measured TCV use at all transplant centers.

Previous studies have investigated the use of 3D-CNN based segmentation approaches for various cardiac applications with cross-sectional imaging. These studies are performed exclusively in adult subjects and focus mainly on individual structures of the heart such as the ventricles, atrium, aorta, scars, blood pool and myocardium [9–11]. We hypothesize that a custom-built 3D-CNN in a Dense-net configuration can calculate TCV from clinical pediatric CT images within an acceptable margin of error for clinical use. The primary goal of this study is the creation of a model that derives TCV from CT scan data in the pediatric population.

## Materials and Methods

### Subject selection

This study was approved by Cincinnati Children’s Hospital Medical Center Institutional Review Board prior to study initiation. Written informed consent of subjects was waived by the Institutional Review Board for the following reasons: 1) All information was retrieved from existing medical records; there was no direct patient contact 2) The study involves no more than minimal risk – No testing, time, risk or procedures beyond those required by routine case were imposed on patients as a result of this study 3) No data beyond that collected in the course of routine care was collected for this study and 4) it was not be possible to contact all patients as the study comprised a large time span. Patients may have been lost to follow-up or deceased. The study was conducted in compliance with the Health Insurance Portability and Accountability Act of 1996 (HIPAA) standards.

A retrospective review of Picture Archiving and Communication Systems (PACS) database was performed to identify pediatric and young adult subjects with normal cardiac anatomy on cross sectional imaging. The imaging types included chest CT with contrast (CT-WC) and without contrast (CT-WO). Subjects with incomplete capture of cardiac structures or any clinically identified cardiac abnormality including nonspecific chamber dilation were excluded. To measure the performance of the model in heart transplant candidates, additional subjects with heart disease listed for heart transplant were included for testing. This included both subjects with cardiomyopathy (CM, Common Data Element Code C34830) and congenital heart disease (CHD, Common Data Element Code C95834). Demographic data was collected via chart review including age, sex, weight, height, diagnosis, and surgical history. Body surface area (BSA) and body mass index were derived from subject height and weight[12].

CTs were performed on an Aquillon One CT scanner (Canon Medical Systems, Otaware, Japan) between January 1, 2010, and January 1, 2020. There was variable slice thickness of the scans (range 0.5–1mm), but most commonly 0.5mm. CT-WC exams included both angiograms and delayed contrast images. CTs were performed as either helical or cone-beam acquisition. Cardiac gating was not routinely performed for all scans. The predominant scan acquisition was “Chest CT,” however, CT with an anatomic range that included neck, chest, abdomen, or pelvis were also included.

### Ground Truth Segmentation

Cross sectional imaging Digital Imaging and Communication in Medicine (DICOM) data were imported into Mimics 3D visualization software (version 23.0, Materialise, Belgium). De-identification was completed within Mimics and ground truth manual segmentation of the TCV was performed, as previously described[6, 7, 13, 14]. In brief, TCV was defined as the segmented myocardial mass and internal heart chamber volumes bounded at the levels of surgical anastomosis for bicaval orthotopic heart transplantation. The TCV measurements included the border of the myocardial mass to the junction of the superior and inferior vena cavae to the right atrial junction, the junction of the pulmonary veins to the left atrium and the level of the aortic and pulmonary roots. The segmentation was performed by a single observer (NAS) with 7 years of segmentation experience. For diseased hearts with atypical anatomy (i.e., Fontan), the TCV segmentation likewise included the entirety of the cardiac mass that would be explanted at time of heart transplantation. Interobserver variability of this segmentation method has previously been reported on as highly reliable[14].

### Export of segmented masks

The Mimics files were de-identified prior to export. The Mimics AI Assistant Plugin (Beta Version) was used to export raw data as NRRD (Nearly Raw Raster Data) file format. Both the raw imaging data and the ground truth segmentation files (i.e. TCV mask) were exported. The NRRD files were then imported into a custom built DenseNet Deep Learning (DL) architecture described below. No resampling or normalization operations were used.

### Training/Validation sets

The dataset was split into a training set (n = 270, 86%) and a validation set (n = 44, 14%). The validation cohort was sized to fairly assess imaging and subject characteristics that may influence model accuracy such as presence of contrast, age, weight, sex.

### Deep Learning Model Training

**Figure 1:**
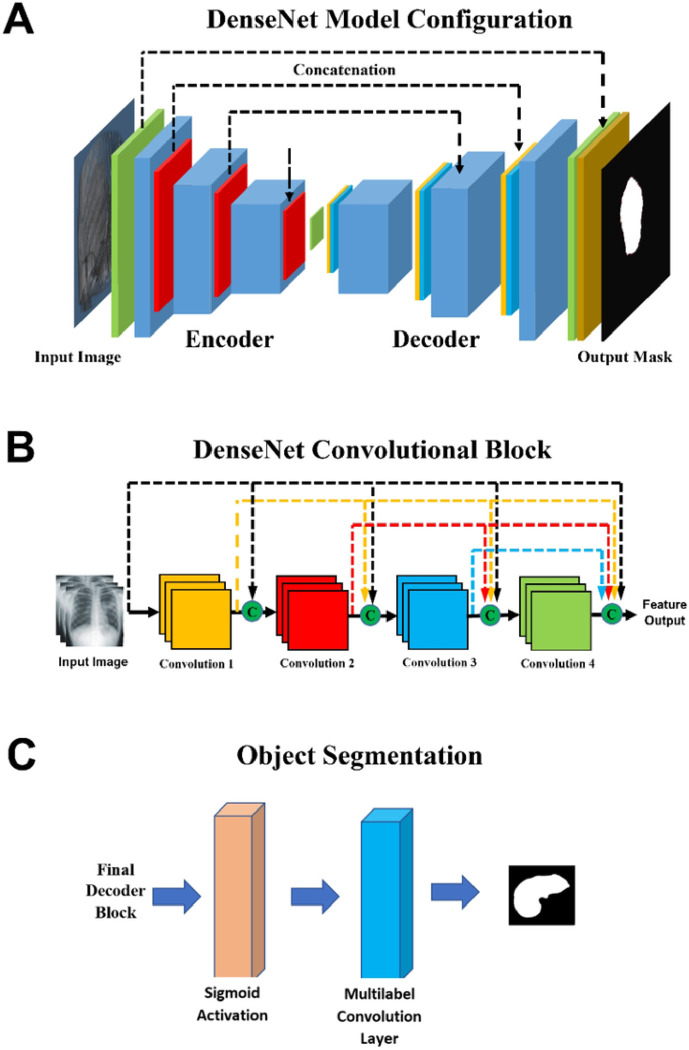
Deep learning architecture components: A) DenseNet encoding section precedes the decoding section. B) ResNet architecture maximized cross connections between convolutional layers preserving the gradients for small features throughout the pipeline C) Sigmoid activation of the final decoder block’s output is fed into a final convolutional layer for multi label object segmentation. TCV, Total Cardiac Volume.

The DenseNet architecture consists of an encoding section followed by a decoding section (Fig. 1a). In the encoding section, spatial information is mapped to feature space by convolutional blocks (blue). In each encoder convolutional block, an image’s spatial volume is reduced while the feature space volume increases. During decoding, deconvolution blocks concatenate features from the previous block and features from the encoding leg to generate the output mask.

For maximum accuracy, the convolution/deconvolution blocks are based on the DenseNet architecture which preserves the gradients for small features throughout the pipeline (Fig. 1b). The DenseNet architecture incorporates maximum cross connections between the blocks’ convolution layers. Each convolutional/deconvolutional layer within a block is followed by a batch normalization and ReLU activation.

A final max pooling of the block’s output is performed before continuing to the next block. Sigmoid activation of the final decoder block’s output is fed into a final convolutional layer for multi label object segmentation Fig. 1c. Dense-Net model training is carried out using ground truth masks generated from training CT images. Model weights are adjusted by batch using the Adams Optimizer with a loss function equal to 50% Dice Loss and 50% Cross Entropy Loss.

### Statistical Analysis

Demographic and clinical characteristics were described using means ± standard deviation for continuous variables and frequencies (percent) for categorical variables. Independent samples t-tests were used to test for differences in normally distributed, continuous variables and Wilcoxon rank-sum tests were used to test for differences in non-normally distributed, continuous variables. The accuracy of the deep learning model was evaluated with the Dice similarity coefficient (DSC), a statistical tool measuring the intersection of voxels of the ground truth and deep learning derived datasets. The deep-learning derived and manually derived TCV segmentation was also used to calculate total cardiac volume. Mean Absolute Percent Error (MAPE), rather than absolute error, was used to compared accuracy of deep learning and manual TCV to allow parity of across the pediatric age range and size. The Fisher’s exact test was used to test for differences in categorical variables. Comparison of normally distributed continuous variables was performed using Pearson correlation. One-way Analysis of Variance (ANOVA) was used to test for differences in continuous variables across three or more groups. Tukey’s post-hoc test was used for pair-wise differences between groups.

## Results

### Demographics

**Figure 2:**
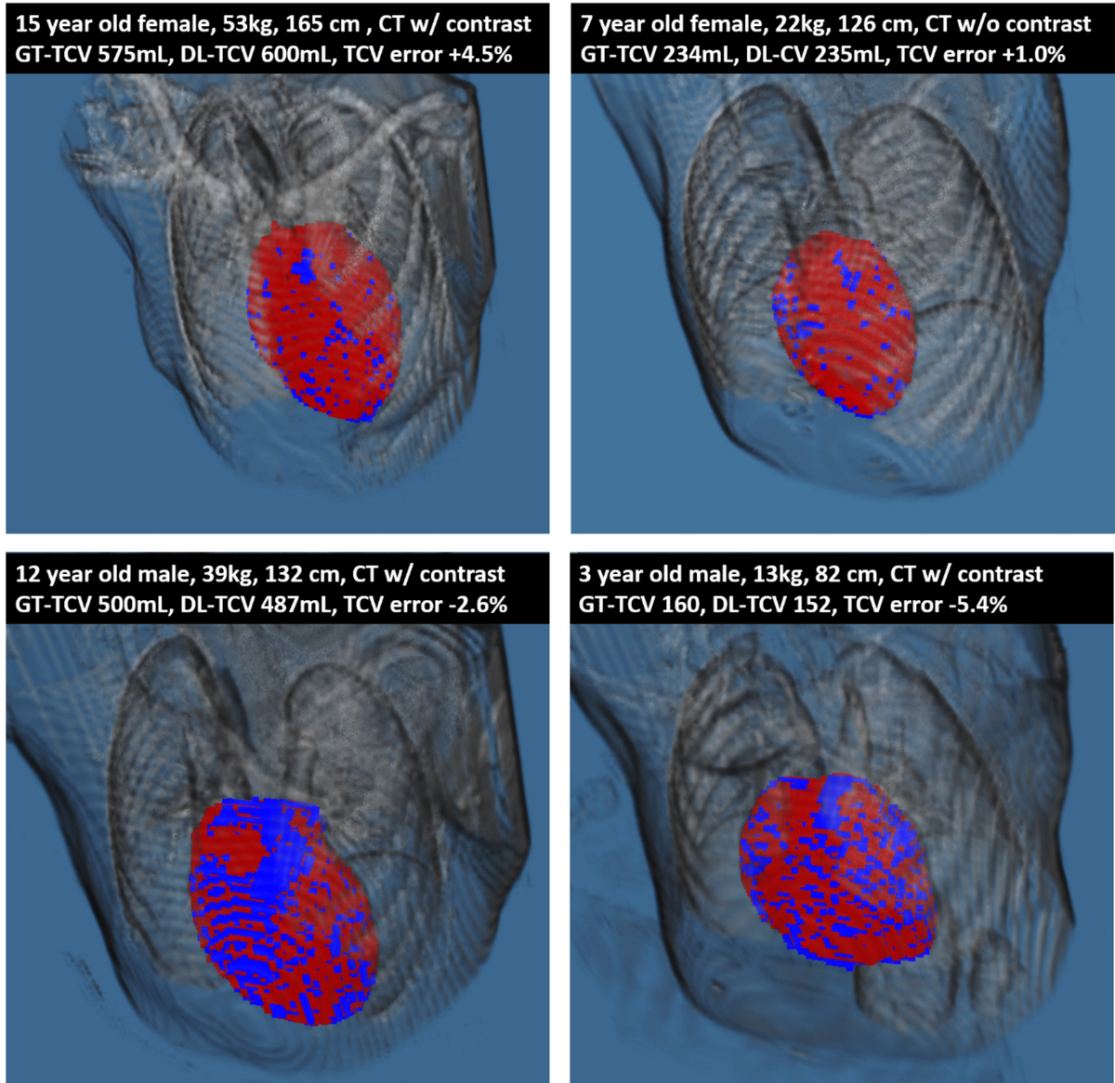
Example Total Cardiac Volume Segmentations (Blue = Ground Truth, Red = Deep Learning). Abbreviations: CT, Computed Tomography; GT-TCV, Ground Truth Total Cardiac Volume; DL-TCV, Deep Learning Total Cardiac Volume

A total of 314 subjects were identified, 168 (54%) of whom were male. The mean weight was 47 ± 34 kg, height was 133 ± 42 cm, body surface area was 1.27 ± 0.64 m^2^, and age was 11 ± 8 years. The mean TCV was 463 ± 287 mL. There are no significant differences between the training and validation groups in weight, height, body surface area, age, sex, or TCV, as summarized in Table 1. Example automatic segmentations are shown in Fig. 2.

The validation group included 44 subjects total, 36 subjects with normal cardiac anatomy. Additionally, 8 subjects with heart failure listed for transplant were included in the validation group, as shown in Table 2. This included 3 subjects with dilated cardiomyopathy (DCM), 1 subject with hypertrophic cardiomyopathy, 2 subjects with failing Fontan physiology, 1 subject listed for re-transplantation, and 1 subject with D-Transposition of the great arteries (D-TGA) status post Senning procedure.

### Model performance

**Figure 3:**
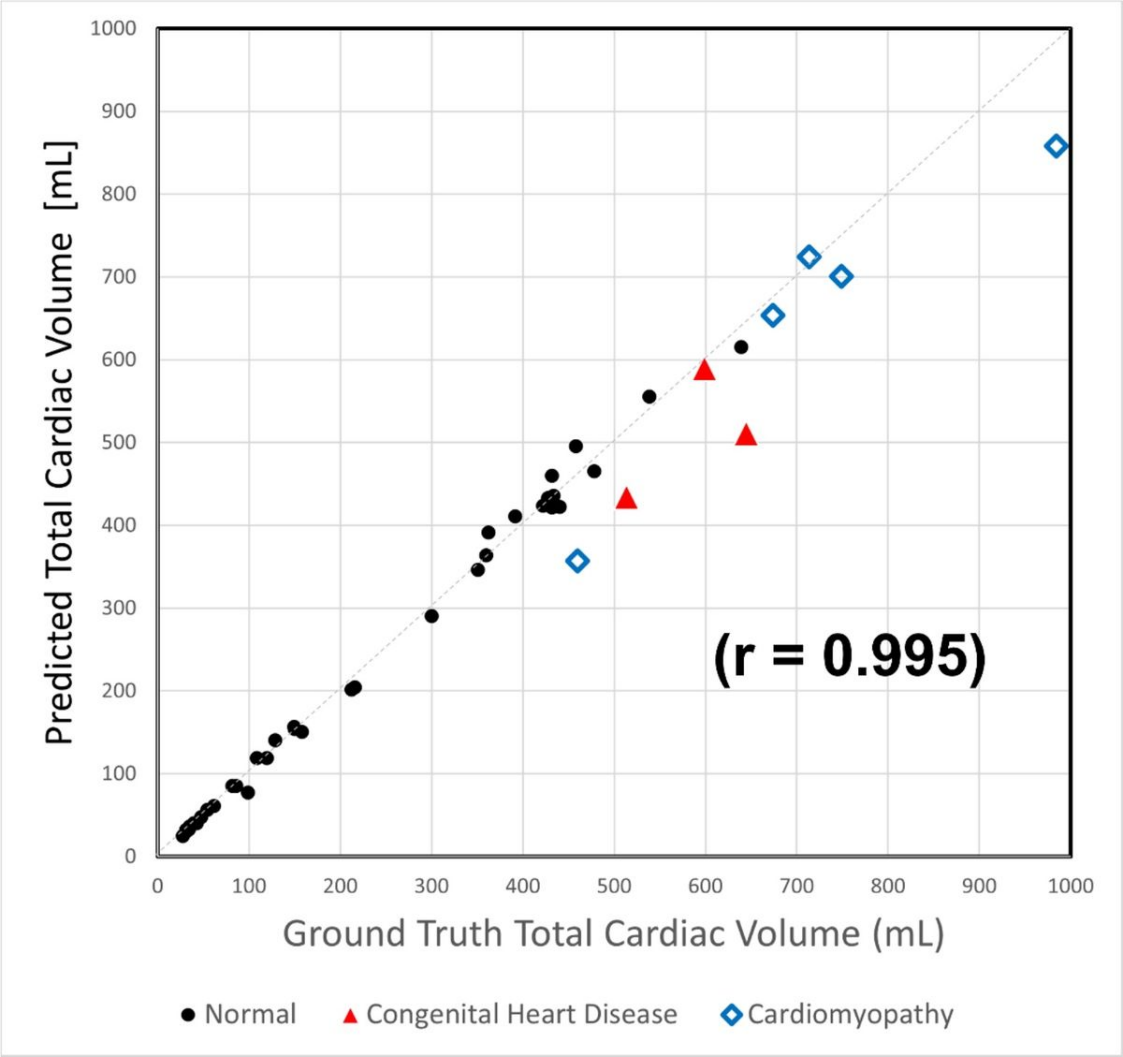
Correlation plot between Ground truth TCV (x axis) and Predicted TCV (y-axis) of the 44 subjects in the validation set. The subgroups are differentiated as follows: black circles (normal cardiac anatomy), red triangles (congenital heart disease) and blue diamonds (cardiomyopathy).

All reported values are for the validation cohort (n = 44). There was excellent correlation between the ground truth and predicted TCV for the validation cohort (r = 0.995), Fig. 3. The mean DSC was 0.94 ± 0.03 (range 0.84–0.97). The mean error in TCV measurement [Error = DL-TCV − Ground Truth TCV] was – 8 mL and mean absolute error of 29 mL. The mean absolute percent error was 5.5%.

### Correlation of DSC to Subject Factors body size, TCV, anatomy

There was no association between DSC and weight (r = 0.12, p = 0.44), height (r = 0.1, p = 0.52), body surface area (r = 0.11, p = 0.48), age (r = 0.01, p = 0.94), sex (p = 0.37), or TCV (r = 0.01, p = 0.93). Subjects with non-contrast CT had a lower average DSC when compared to CT with contrast (0.93 vs. 0.94), but this did not reach statistical significance (p = 0.2).

### Increased Error in CHD scans

**Figure 4:**
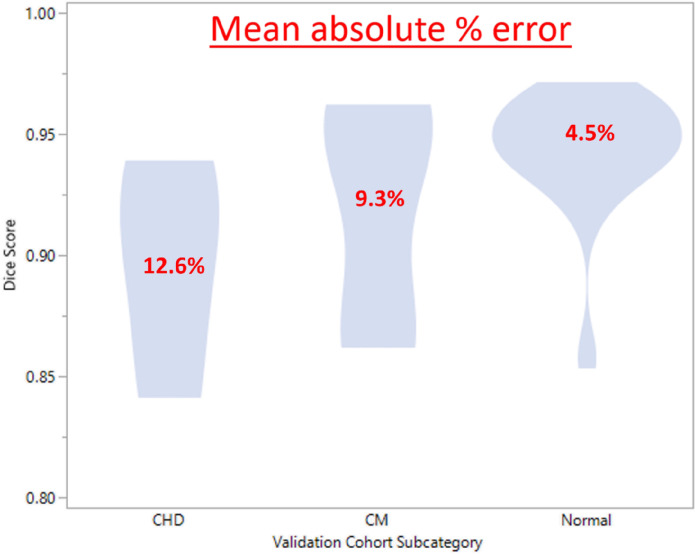
Violin plot demonstrating the Dice similarity Coefficient (DSC) among subsets of subjects within the validation cohort. The average DSC is highest for the normal cohort, densely concentrated above 0.90. The cardiomyopathy (CM) and congenital heart disease (CHD) cohorts are more uniformly distributed with lower dice score than the normal cohort.

The mean DSC was 0.90 [95% CI 0.86–0.93], 0.92 [95% CI 0.89–0.95], and 0.94 [95% CI 0.93–0.95] for the CHD, CM, and normal cohort, respectively (ANOVA omnibus p = 0.017; post-hoc Tukey’s test normal vs. CHD p = 0.033, normal vs. CM 0.08, CM vs. CHD 0.09). The mean absolute percent error in TCV measurement was 12.6% [95% CI 6.7–18.4%], 9.3% [95% CI 4.7–13.8%], and 4.5% [95% CI 2.7–6.1%], for the CHD, CM, and normal cohort (p = 0.009), Fig. 4.

Bias towards under-prediction of TCV in those with “abnormal” anatomy.

The DL-TCV of subjects in the pre-transplant cohort underestimated the ground truth TCV. On average, the DL-TCV prediction was approximately 10% below the ground truth TCV.

## Discussion

To our knowledge, this is the first study to demonstrate that TCV can be automatically measured from a CT scan using a DL model in the pediatric population and in subjects with congenital heart disease. Prior studies evaluating deep learning methods for automated calculation of TCV have been limited to adult populations without cardiac disease[11].

### AI model performance

The DL-TCV showed a strong correlation with the ground truth TCV which was also demonstrated by high DSC with low percent error. The estimated TCV values determined by the TCV model were highly accurate, with an absolute percent error of < 10% for all subjects except one. This fits within our current benchmark error value of 10%, which is approximately the expected variation in TCV throughout the cardiac cycle[15]. The DSC of 0.94 is comparable to the DSC of 0.96 reported by Shahzad et al. using a multi-atlas technique in a healthy adult population[11]. However, the study by Shahzad et al is significantly limited by use of a computationally derived reference standard as the ground truth, rather than manual segmentation. To our knowledge, no other studies have reported on the accuracy of deep learning TCV segmentation.

From a technical standpoint, the performance of the DenseNet DL model is mostly governed by the internal design of the convolution/deconvolution blocks which enable the model to ascertain fine features without overfitting. DenseNet architecture contains multiple connections between layers that preserves spatial information while minimizing the number of parameters within the model, thus enabling it to be trained on a relatively small number of scans.

### AI performance in pre-transplant

The DL predicted TCV errors were slightly larger in the pre-transplant (i.e. heart disease) population. In general, the DL model underestimated the TCV in those with CHD and those with cardiomyopathies. This is likely because the DL model was trained predominantly on normal subject CT scans. However, in real world setting, such a recipient would likely require manual segmentation. The donor can be determined via automated segmentation through the process described above. In future studies, models trained with a more balanced dataset of disease and healthy subjects may produce a model usable for both donor and recipient.

### Clinical Use

A DL model for TCV has an immediate practical use in pediatric heart transplantation. Over the past few years, the United Network of Organ Sharing (UNOS) has developed a secure cloud-based imaging hub for sharing donor imaging data[16]. The DL model could automatically measure the TCV of the uploaded CT scans for clinical comparison by the recipient transplant team. Employing a DL model on the image hub would enable the transplant team to perform an immediate donor size match test using donor imaging without the need to perform a manual segmentation, which is not available at most centers.

Before the implementation of such a system, the generalizability and robustness of the DL TCV segmentation model must be demonstrated. A federated learning approach would enable training and testing of a DL model using data from multiple institutions. Federated learning is a method by which several models are trained simultaneously at various sites and the model weights are periodically aggregated and redistributed[17]. Since only the learned model weights are shared between institutions during federated learning, patient data does not need to be shared. The model detailed in this manuscript has laid the groundwork for a future multi-institutional federated learning TCV model.

In addition to the potential applications within the transplant population, this work demonstrates a pathway to use imaging data labeled for clinical purposes to train an AI model. This potentially opens the door to using scans pre-labeled for clinical use to train AI models (i.e. data does not need to be relabeled in a research setting), saving time and resources.

### Limitations

This study carries the typical limitations of a single-center study. The deep learning model was trained on a limited number of subjects with heart disease, limiting its current application in this population. The number of scans available in this single institution was adequate to train a model, though a future multicenter study may have the advantage of more scans with congenital pathology to enable more accurate TCV measurement.

## Conclusion

TCV on CT can be used to match the size of the donor and recipients for heart transplant, however segmentation is labor intensive and impractical. This study finds that a 3D convolution neural network can automatically and accurately measure TCV with a mean absolute error of 5.5%.

## Figures and Tables

**Table 1:** Demographics of Training and Validation Datasets

	Total (n = 314)	Training (n = 270)	Validation (n = 44)	
	mean ± SD	mean ± SD	mean ± SD	p value
Weight (kg)	47 ± 34	47 ± 34	47 ± 36	0.95
Height (cm)	133 ± 42	134 ± 41	131 ± 43	0.73
BSA (m^2^)	1.27 ± 0.64	1.27 ± 0.64	1.25 ± 0.68	0.85
Age (years)	11 ± 8	11 ± 8	11 ± 9	0.92
TCV (mL)	463 ± 287	457 ± 279	498 ± 334	0.44
Sex = M	168 (54%)	146 (54%)	22 (50%)	0.62

**Table 2:** Metrics of Model Performance. Data partitions of Normal and Pre-transplant cohorts are also included. Abbreviations: DSC, Dice similarity coefficient; TCV, Total Cardiac Volume.

	All n = 44)	Normal (n = 36)	Pre-Transplant (n = 8)	p value
DSC	0.93 ± 0.04	0.94 ± 0.03	0.91 ± 0.05	0.09
Loss (%)	3.8 ± 2.1	3.3 ± 1.6	6.0 ± 3.2	0.05
TCV: error [mL]	−8 ± 47	5.8 ± 25.6	−70.1 ± 68.3	0.02
TCV: Absolute error [mL]	29 ± 36	18 ± 19	76 ± 62	0.03
TCV: % error (%)	1.9 ± 7.6	0.06 ± 5.9	−10.1 ± 9.0	0.02
TCV: Absolute % error (%)	5.5 ± 5.5	4.5 ± 3.9	10.5 ± 8.5	0.08

## Data Availability

The datasets generated during and/or analyzed during the current study are available from the corresponding author on reasonable request. The full study protocol is available upon reasonable request to the corresponding author.
